# T2 New York City Structural Fires are Linked to Inequities in Safe Heating for Immigrant Communities

**DOI:** 10.1093/jbcr/irad045.002

**Published:** 2023-05-15

**Authors:** Eloise Stanton, Clifford C Sheckter, Julia Biedry, Danielle Rochlin

**Affiliations:** Keck School of Medicine of USC, LOS ANGELES, California; Stanford University, Palo Alto, California; Tableau Software, New York, New York; Stanford University, Palo Alto, California; Keck School of Medicine of USC, LOS ANGELES, California; Stanford University, Palo Alto, California; Tableau Software, New York, New York; Stanford University, Palo Alto, California; Keck School of Medicine of USC, LOS ANGELES, California; Stanford University, Palo Alto, California; Tableau Software, New York, New York; Stanford University, Palo Alto, California; Keck School of Medicine of USC, LOS ANGELES, California; Stanford University, Palo Alto, California; Tableau Software, New York, New York; Stanford University, Palo Alto, California

## Abstract

**Introduction:**

The US was rattled January 9th, 2022 when a structural fire claimed the lives of eight children and nine adults in New York City’s (NYC) Bronx borough. Existing reports suggest that in low-income historically Black/Latinx communities, heating complaints are often ignored. While this pattern implies housing inequities, landlord negligence, and greater risk of residential fires in the Bronx and other low socioeconomic neighborhoods, this postulation has not been scientifically evaluated. We investigated the relationship between structural fires and heating complaints in NYC, leveraging municipal data from 59 community districts.

**Methods:**

Data from the NYC Open Data Portal Fire Incident Dispatch data and Heat/Hot Water Complaints were merged to identify the number of heating complaints and structural fires per month in each NYC community district from 2017-2022. Population and demographic comparisons were made using US Census–NYC Demographics 2020 data. The primary outcome was number of structural fires per month which was modeled with mixed effects multivariable regression. Community districts were treated as random intercepts given demographic and architectural variation between districts. Statistical analyses were performed using Stata/IC Version 17.0 (StataCorp LLC).

**Results:**

Within NYC’s 59 community districts, 3,877 heating complaints were filed against 3,989 structural fires during the study period (Figure 1). The mixed effects model demonstrated a significant association between heat complaints and frequency of structural fires (coefficient 0.013, 95% CI .012-.014 p< .001,). Further, the model demonstrated significant variance among community districts in predicting structural fires (σ2=215, p < .001). The Likelihood ratio test comparing the mixed effects linear model to a simple linear regression yield a p-value of < 0.001 in favor of the mixed effects model.

**Conclusions:**

The frequency of heating complaints was significantly associated with the frequency of structural fires in NYC. Importantly, this association varied across community districts with more fires occurring in districts with greater proportions of Black/Latinx residents. Our results highlight a pattern adversely affecting marginalized racial/ethnic communities in which ongoing ignored heating complaints and landlord negligence may lead residents to use unsafe heating practices, inadvertently creating structure fires, morbidity, and death.

**Applicability of Research to Practice:**

Our findings can help guide and educate both clinicians and policymakers in regard to socioeconomic disparities and epidemiological trends implicated in burn care.

